# Serial changes in tumour measurements and apparent diffusion coefficients in prostate cancer patients on active surveillance with and without histopathological progression

**DOI:** 10.1259/bjr.20210842

**Published:** 2021-09-19

**Authors:** Nikita Sushentsev, Iztok Caglic, Leonardo Rundo, Vasily Kozlov, Evis Sala, Vincent J Gnanapragasam, Tristan Barrett

**Affiliations:** 1 Department of Radiology, Addenbrooke’s Hospital and University of Cambridge, Cambridge, UK; 2 Cancer Research UK Cambridge Centre, University of Cambridge, Cambridge, UK; 3 Department of Public Health and Healthcare Organisation, Sechenov First Moscow State Medical University, Moscow, Russia; 4 Division of Urology, Department of Surgery, University of Cambridge, Cambridge, UK; 5 Cambridge Urology Translational Research and Clinical Trials Office, University of Cambridge, Cambridge, UK

## Abstract

**Objective:**

To analyse serial changes in MRI-derived tumour measurements and apparent diffusion coefficient (ADC) values in prostate cancer (PCa) patients on active surveillance (AS) with and without histopathological disease progression.

**Methods:**

This study included AS patients with biopsy-proven PCa with a minimum of two consecutive MR examinations and at least one repeat targeted biopsy. Tumour volumes, largest axial two-dimensional (2D) surface areas, and maximum diameters were measured on *T*_2_ weighted images (*T*_2_WI). ADC values were derived from the whole lesions, 2D areas, and small-volume regions of interest (ROIs) where tumours were most conspicuous. Areas under the ROC curve (AUCs) were calculated for combinations of *T*_2_WI and ADC parameters with optimal specificity and sensitivity.

**Results:**

60 patients (30 progressors and 30 non-progressors) were included. In progressors, *T*_2_WI-derived tumour volume, 2D surface area, and maximum tumour diameter had a median increase of +99.5%,+55.3%, and +21.7% compared to +29.2%,+8.1%, and +6.9% in non-progressors (*p* < 0.005 for all). Follow-up whole-volume and small-volume ROIs ADC values were significantly reduced in progressors (−11.7% and −9.5%) compared to non-progressors (−6.1% and −1.6%) (*p* < 0.05 for both). The combined AUC of a relative increase in maximum tumour diameter by 20% and reduction in small-volume ADC by 10% was 0.67.

**Conclusion:**

AS patients show significant differences in tumour measurements and ADC values between those with and without histopathological disease progression.

**Advances in knowledge:**

This paper proposes specific clinical cut-offs for *T*_2_WI-derived maximum tumour diameter (+20%) and small-volume ADC (−10%) to predict histopathological PCa progression on AS and supplement subjective serial MRI assessment.

## Introduction

Active surveillance (AS) is the recommended management option for patients with low- and intermediate-favourable risk prostate cancer (PCa), who account for nearly half of the newly diagnosed cases in the US and the UK.^1–4^ During the first 5 years on AS, 27.5% of patients switch to radical treatment due to disease progression, with a further 12.8% leaving AS programmes for other reasons, including anxiety and concerns related to the invasive nature of repeat biopsies.^5^ Repeat biopsies do remain the cornerstone of clinical decision-making and the switch to radical treatment; however, they also present a key barrier to patient uptake of and adherence to AS.^2,6,7^ Therefore, reducing the need for unnecessary biopsies during AS presents an important clinical challenge.

To address this, many centres have increased their reliance on MRI to navigate treatment decisions without a mandatory follow-up histopathological assessment.^8^ According to two recent meta-analyses,^9,10^ serial MRI has a reasonable pooled negative predictive value of up to 0.81; however, the maximum pooled positive predictive value for detecting histopathological disease progression to grade group ≥2 is 0.52, highlighting the inability of serial MRI to completely replace repeat biopsies as part of AS follow-up.^11,12^

There are several factors that may explain the limitations of MRI in the context of AS. These include considerable variability of MR imaging quality^13,14^ and radiologists’ experience,^15,16^ as well as the subjective nature of the Prostate Cancer Radiological Estimation of Change in Sequential Evaluation (PRECISE) recommendations^17^ designed to standardise serial MRI reporting on AS. More specifically, PRECISE assessment criteria lack a clear definition of a “clinically significant” radiological progression, which is particularly critical for assigning a PRECISE category 4, defined as “significant increase in size and/or conspicuity”. Consequently, PRECISE scoring shows no superiority over non-standardised institutional criteria of disease progression,^9,10^ highlighting an unmet clinical need for providing objective MRI biomarkers in the AS setting.^18^ While several attempts have been made to identify specific tumour measurements and apparent diffusion coefficient (ADC) cut-offs that could offer quantitative surrogates of “clinically significant” radiological disease progression,^19–22^ these studies lacked systematic histopathological assessment at follow-up, which limits the reliability of their findings given the potential limitations of MRI changes *vs* gold-standard histopathology.

In this study, we applied several common segmentation approaches to analyse serial changes in MRI-derived tumour measurements and ADC values in AS patients with and without histopathological PCa progression. Thus, we aimed to identify objective clinically applicable cut-off values that could be used in the follow-up assessment of MR-visible lesions in patients with PCa on AS.

## Methods

### Patient population

**Figure 1.**
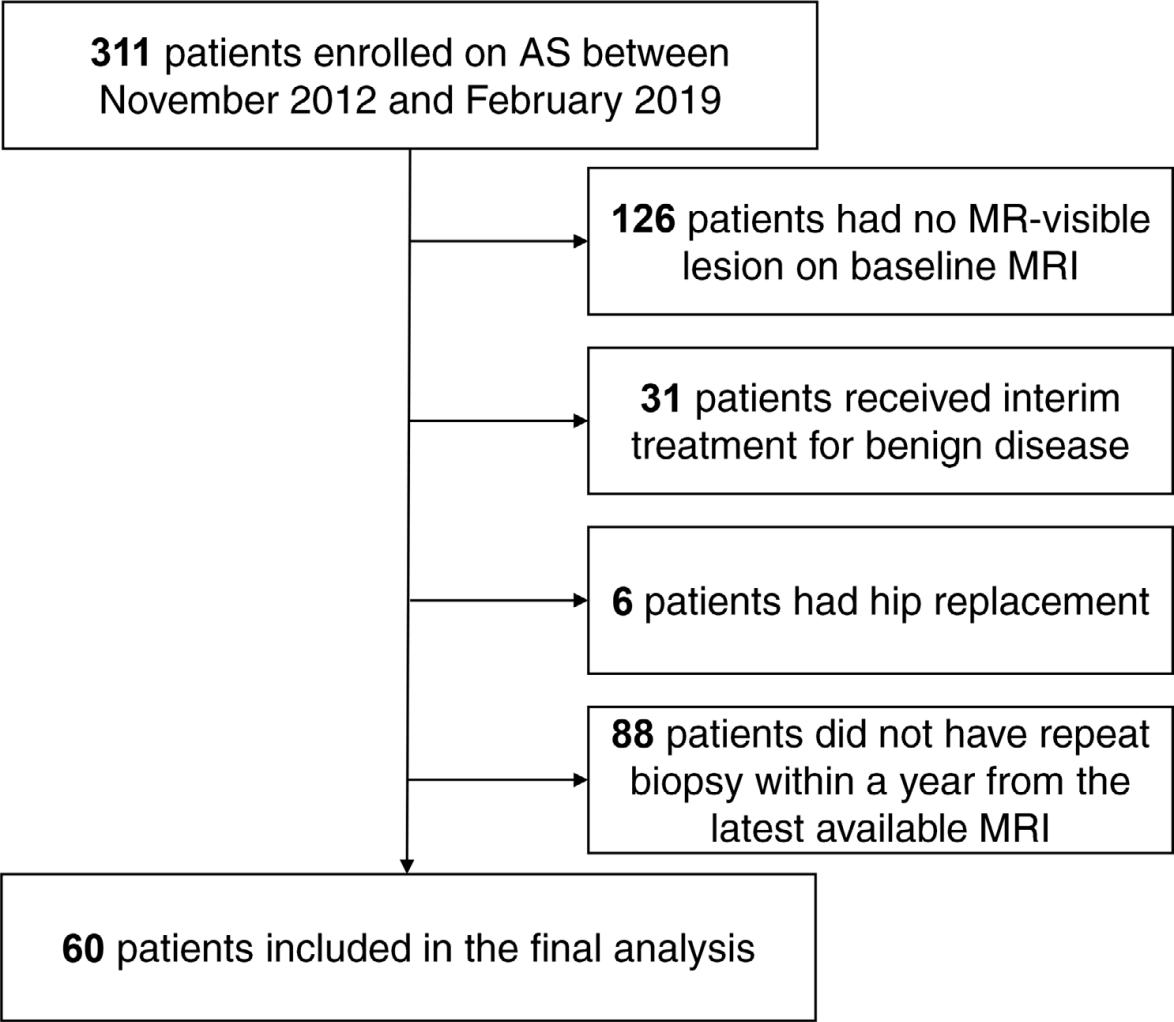
Study flow chart.

This retrospective cohort study was approved by the local Institutional Review Board (reference number: HBREC.2020.49). The study included consecutive patients with biopsy-proven PCa enrolled on the local AS programme according to the eligibility criteria reported previously.^23^ The inclusion criteria were the presence of an MR-visible lesion at baseline, AS follow-up length of at least 2 years with at least two MRI examinations performed on the same 3 T magnet, and at least one repeat targeted biopsy within 12 months of the most recent MRI. The exclusion criteria were any prior or interim treatment for PCa or benign disease, or the presence of any pelvic metalwork. The study flowchart presented in Figure 1 summarises the patient selection process.

60 patients meeting inclusion criteria and enrolled on AS in our centre between August 2013 and May 2018 were included in the final analysis and divided into two groups depending on their disease progression status. Histopathological AS progression (*n* = 30) was defined as grade group progression on repeat targeted biopsy. The control group (*n* = 30) included patients harbouring both radiologically (highest PRECISE score 1–3^24^ over the course of AS) and histopathologically stable disease confirmed as part of routine repeat biopsies mandated by the local AS protocol (see *Biopsy technique* section).

### MRI technique

Patients underwent prostate MRI on a 3 T MR750 system (GE Healthcare, Waukesha, WI) using a 32-channel receiver coil. Intravenous injection of hyoscine butylbromide (Buscopan, 20 mg ml^−1^; Boehringer Ingelheim, Ingelheim am Rhein, Germany) was administered prior to imaging unless clinically contraindicated. Multiparametric MRI protocol included axial *T*_1_ weighted imaging, multiplanar high-resolution *T*_2_ weighted two-dimensional (2D) fast recovery fast spin echo (FSE), spin-echo echoplanar imaging pulse diffusion-weighted imaging (DWI), and dynamic contrast-enhanced (DCE) imaging, as described previously.^25^ DWI was performed with b-values: b-150, b-750, and b-1,000; with additional small field of view DWI obtained using b-2,000s/mm^2^; ADC maps were automatically calculated. Follow-up studies did not include post-contrast DCE sequences, but the protocol was otherwise identical for all patients and all scans included in the analysis.

### Biopsy technique

Targeted biopsy was performed using MRI/ultrasound fusion by either a transrectal (TR, DynaCAD, InVivo Corp, Orlando, FL) or transperineal (TP, Biopsee, Oncology Systems Limited, Shrewsbury, UK), with 2–4 target cores and 12 (TR) or 24 (TP) systematic cores background cores, as previously described.^23^ Repeat targeted biopsies were either performed at time points specified by the local protocol (12 and 36 months), or triggered earlier by clinical suspicion of progression, defined as either three consecutive elevated PSA levels above the pre-defined threshold or suspected radiological progression (PRECISE scores 4–5).

### Image segmentation and analysis

**Figure 2.**
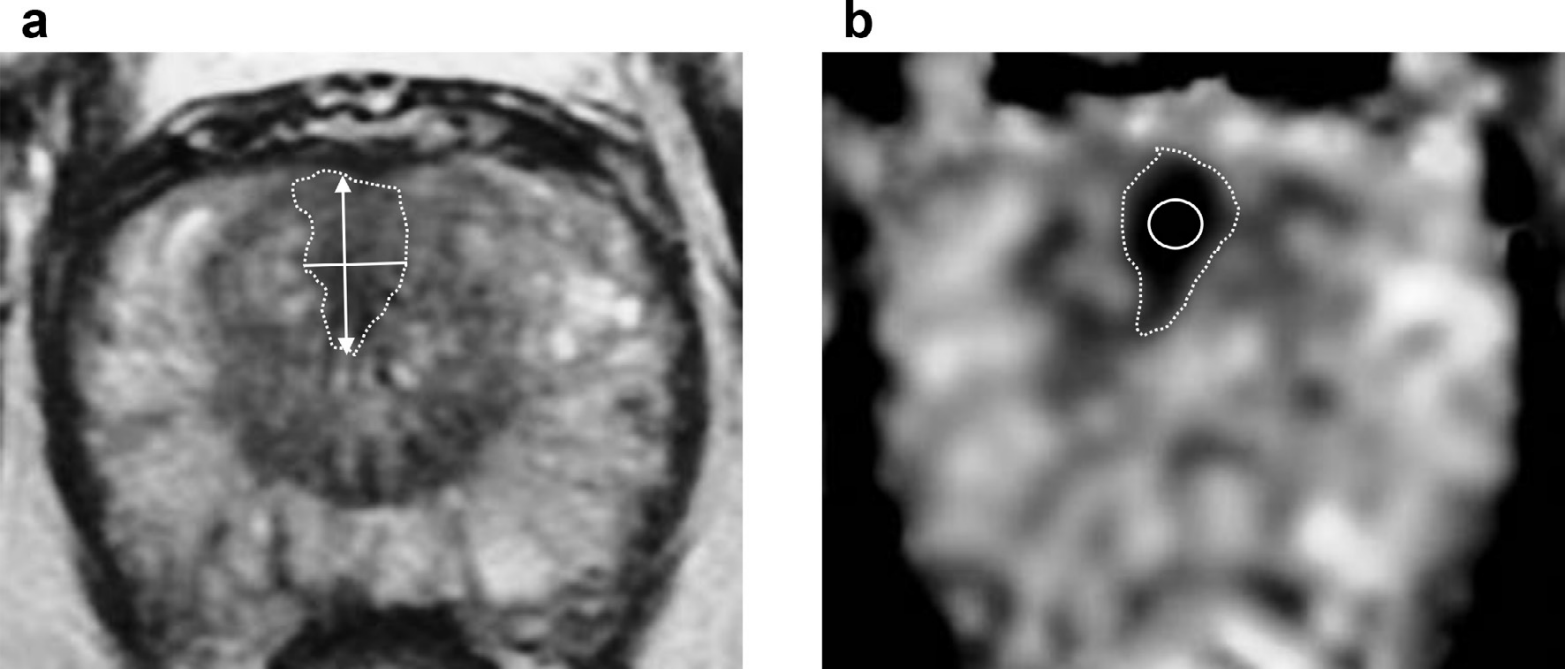
Example axial *T*_2_WI (**A**) and ADC map (**B**) derived from slices where the outlined anterior transition zone lesion demonstrated its maximum diameter. Free-hand ROIs delineating the lesion were additionally drawn on all slices encompassing the tumour and were used to measure *T*_2_WI- and ADC-derived tumour volumes, as well as whole-tumour ADC values. Image (**A**) illustrates linear measurements of *T*_2_WI-derived maximum tumour diameter (arrow), and a second perpendicular short-axis diameter (white line) used to calculate 2D tumour surface area. Image (**B**) demonstrates an example free-hand ROI from which mid-slice ADC values were derived, along with a uniform small ROI (white circle) used to obtain small-volume ADC. 2D, two-dimensional; ADC, apparent diffusion coefficient; ROI, region of interest; *T*_2_WI, *T*_2_ weighted imaging

Tumour regions of interest (ROIs) were drawn on anatomical *T*_2_ weighted images and on ADC maps (Figure 2) in consensus by a fellowship-trained uroradiologist (TB) with 13 years’ experience of reporting prostate MRI and an imaging research fellow (NS) with 4 years’ experience using open-source software ITK-SNAP.^26^ Image quality was adequate in all cases for reliable lesion delineation on both *T*_2_WI and ADC maps.

The index lesions^27^ were measured on both *T*_2_WI and ADC maps using the following techniques: (i) volumetric, with tumour segmentation by means of free-hand delineation on all slices encompassing the lesion, (ii) 2D tumour surface area derived using the largest diameter and a second perpendicular diameter ([anteroposterior diameter × transverse diameter × π]/2), and (iii) single maximum tumour diameter (Figure 2).

Tumour-derived ADC values were measured using the following techniques: (i) whole-volume ADC derived from ROIs used to measure tumour volume, (ii) mid-slice ADC derived from a free-hand ROI encompassing the largest tumour area from the axial acquisition used to measure 2D surface area and single maximum diameter, (iii) small-volume ADC obtained from a standardised circular ROI (minimum ROI surface area 15 mm^2^) positioned in the centre of the lesion on a slice where it was most conspicuous (Figure 2).

At follow-up, PRECISE scores were assigned by three subspecialist uroradiologists (IC, ES, TB) with 5–16 years’ experience of reporting prostate MRI and considered to be experts with each having read >2000 cases.^13,28^ The readers were not blinded to clinical information, including PSA and PSA density dynamics. PRECISE scores were assigned prospectively in all cases enrolled in the study after June 2016; for studies performed prior to that date, the scores were applied retrospectively in consensus.

### Statistical analysis

Statistical analyses were conducted using GraphPad Prism (v. 9.0.2, GraphPad Software, San Diego, CA) and SPSS Statistics 17.0 (IBM Corporation, Armonk, NY). Normal distribution of the data was assessed using the D’Agostino-Pearson test (threshold *p*-value ≥ 0.05). To evaluate the relationship between *T*_2_WI- and ADC-derived tumour measurements, we used the Spearman’s rank correlation test, with the agreement between the two sequences assessed using the Bland–Altman test. Intergroup differences in tumour measurements and ADC values between histopathological progressors and non-progressors were measured using the Mann–Whitney *U* test. A subanalysis was also performed in patients with radiological disease progression (*n* = 27, at least one MRI had PRECISE score of 4–5) and with radiologically stable disease (*n* = 33, all MRIs had PRECISE scores 1–3). All statistical tests were two-sided, and *p*-values below 0.05 were considered significant. The receiver operating characteristic (ROC) analysis was performed, with the resulting areas under the ROC curve (AUCs) derived for relative changes in tumour measurements and ADC values obtained from the baseline and final MRI examinations. The DeLong test was used to compare the differences between individual AUCs.

## Results

60 patients were included in the study, with a median age of 67 years (interquartile range 59–70) and PSA of 5.6 ng ml^−1^, with a median follow-up of 45 months (IQR, 30–51 months); Table 1. Patients without progression (*n* = 30) were followed up for significantly longer time at 48 months, compared to patients with histopathological progression (*n* = 30) at 36 months (*p* = 0.002). Baseline PSA and PSA density were significantly higher in progressors (*p* = 0.01 and 0.001, respectively), a difference that became even more pronounced at follow-up (*p* < 0.001 for both). 45/60 (75%) had ISUP Grade Group 1 and 15/60 (25%) patients Group 2 disease at enrolment. 44/60 (73%) and 16/60 (27%) of index lesions were located in the PZ and TZ of the prostate, respectively. Of the 30 patients who showed histopathological progression, 16 were treated with hormone and radiotherapy, 8 underwent prostatectomy, and 6 had brachytherapy.

**Table 1.** Summary baseline clinicopathological characteristics of the study cohort

Parameter	Total cohort (*n* = 60)	Progressors (*n* = 30)	Non-progressors (*n* = 30)	*p*-value
**Age, years**	67 (59–70)	67 (60–70)	68 (59–69)	0.79
**Baseline gland volume, mL**	45.0 (33.3–55.8)	45 (28–52)	45 (37–58)	0.47
**Baseline PSA, ng/mL**	5.6 (3.7–7.7)	6.7 (4.3–8.7)	4.8 (3.1–6.6)	0.01
**Baseline PSA density**	0.12 (0.09–0.19)	0.17 (0.10–0.17)	0.10 (0.07–0.17)	0.001
**Follow-up PSA, ng/mL**	7.4 (5.4–11.0)	9.9 (6.7–12.2)	6.1 (4.2–8.1)	0.0007
**Follow-up PSA density, (ng/mL)/mL**	0.15 (0.10–0.22)	0.21 (0.15–0.25)	0.12 (0.08–0.16)	0.0002
**AS follow-up, mo**	45 (30–51)	36 (26–48)	48 (39–63)	0.002
**Biopsy grade Group 1** **(3 + 3=6), n (% total)**	45 (75%)	24 (80%)	21 (70%)	-
**Biopsy grade Group 2 (3 + 4=7), n (% total)**	15 (25%)	6 (20%)	9 (30%)	
**Target lesion in the PZ, n (% total)**	44 (73%)	19 (63%)	25 (83%)	-
**Target lesion in the TZ, n (% total)**	16 (27%)	11 (37%)	5 (17%)	

PSA, prostate-specific antigen; PZ, peripheral zone; TZ, transition zone.

The data are presented as the median (interquartile range) unless indicated otherwise. The *p*-values are presented for an intergroup comparison between progressors and non-progressors performed using the Mann–Whitney *U* test.

### Relationship between *T*_2_WI- and ADC-derived tumour measurements

Significant positive correlations were observed between *T*_2_WI- and ADC-derived measurements of tumour volume (r_s_ = 0.92), 2D surface area (0.75), and maximum diameter (0.67) for all lesions (*p* < 0.0001 for all). There was also acceptable agreement between *T*_2_WI- and ADC-derived tumour measurements for PZ and TZ lesions when separately obtained according to the PI-RADS dominant zonal sequence paradigm^29^ (Spearman’s correlation and Bland–Altman analyses in Supplementary Material 1).

Supplementary Material 1.

### Comparison of tumour measurements in progressors *vs* non-progressors

At baseline, progressors and non-progressors demonstrated no significant differences between *T*_2_WI-derived tumour volume, 2D surface area, and maximum tumour diameter (Table 2). In progressors, at follow-up MRI all *T*_2_WI-derived tumour measurements were significantly higher (*p*-value range, 0.004–0.007). Conversely, in non-progressors, no significant changes were noted between any of the *T*_2_WI-derived tumour measurements at follow-up MRI scans (Table 2). Notably, in progressors, *T*_2_WI-derived tumour volume, 2D surface area, and maximum tumour diameter had a median relative follow-up increase of 99.5%, 55.3%, and 21.7% compared to 29.2%, 8.1%, and 6.9% in non-progressors (*p* =<0.0001,<0.0001, and 0.003, respectively) (Table 2). Box-and-whisker plots illustrating these results are presented in Supplementary Material 1.

**Table 2.** *T*_2_WI-derived tumour measurements obtained from baseline and latest available follow-up MRI scans in patients on active surveillance

Parameter	Total cohort (*n* = 60)	Progressors (*n* = 30)	Non-progressors (*n* = 30)	*p*-value
***T*_2_WI-derived tumour volume (mm^3^)**				
**Baseline MRI**	425.5 (228.4–814.9)	497.7 (277.4–879.7)	320.8 (211.4–638.9)	0.21
**Follow-up MRI**	659.3 (312.5–1424.0)	875.5 (524.8–1947.0)	463.4 (252.5–772.5)	0.005
*p*-value **(baseline *vs* follow-up)**	-	0.007	0.20	-
**Relative change (%) (baseline *vs* follow-up)**	53.5 (19.7–106.7)	99.5 (34.3–160.7)	29.2 (3.7–60.7)	<0.0001
***T*_2_WI-derived 2D surface area (mm^2^)**				
**Baseline MRI**	50.7 (30.7–86.1)	50.7 (33.1–89.9)	50.7 (24.9–80.9)	0.37
**Follow-up MRI**	66.3 (37.5–105.9)	89.4 (66.5–131.0)	40.3 (30.2–68.2)	0.0003
*p*-value **(baseline *vs* follow-up)**	-	0.004	0.94	-
**Relative change (%) (baseline *vs* follow-up)**	26.2 (1.0–70.2)	55.3 (25.1–109.9)	8.1 (-15.9–8.5)	<0.0001
***T*_2_WI-derived maximum tumour diameter (mm)**				
**Baseline MRI**	13.0 (9.4–15.6)	13.3 (9.1–15.6)	12.9 (9.8–15.8)	0.96
**Follow-up MRI**	16.0 (12.2–19.5)	17.0 (13.5–20.8)	14.9 (10.7–17.9)	0.12
*p*-value **(baseline *vs* follow-up)**	-	0.005	0.17	-
**Relative change (%) (baseline *vs* follow-up)**	17.1 (2.9–34.8)	21.7 (10.1–50.0)	6.9 (-1.4–22.2)	0.003

2D, two-dimensional; *T*_2_WI, *T*_2_ weighted imaging.

The data are presented as median (interquartile range). The *p*-values were derived using the Mann–Whitney *U* test and are presented for intergroup comparisons between the absolute *T*_2_WI-derived measurements obtained from progressors and non-progressors, baseline and follow-up scans in patients from the same groups, as well as between relative changes in the measurements derived from baseline and follow-up MRI scans

### Comparison of tumour ADC values in progressors *vs* non-progressors

Similar to *T*_2_WII-derived tumour measurements, no significant baseline intergroup differences were noted between whole-volume, mid-slice, and small-volume ADC values derived from tumours in patients with and without histopathological disease progression (Table 3). In non-progressors, ADC values measured using all three techniques did not change at follow-up compared to baseline (*p*-value range, 0.08–0.71), while progressors demonstrated a significant follow-up decrease in all tumour-derived ADC values (*p*-value range, 0.0009–0.03) (Table 3). Interestingly, the relative change in the median mid-slice ADC values between progressors and non-progressors was non-significant (-9.5% vs  5.6%, respectively; *p* = 0.79). Conversely, the relative changes in the follow-up median whole-volume and small-volume ADC values were significantly greater in progressors compared to non-progressors (−11.7% and −9.5vs−6.1% and −1.6%, respectively; *p* = 0.02 and 0.008, respectively) (Table 3). These results are illustrated in Supplementary Material 1.

**Table 3.** Tumour ADC values derived from baseline and follow-up MRI scans in active surveillance patients

Parameter	Total cohort (*n* = 60)	Progressors (*n* = 30)	Non-progressors (*n* = 30)	*p*-value
**Whole-volume ADC, 10^−6^ mm^2^/s**				
**Baseline MRI**	882.9 (811.7–1002.0)	863.8 (812.2–957.4)	893.1 (800.8–1041.0)	0.43
**Follow-up MRI**	811.3 (738.5–892.4)	786.6 (732.1–841.4)	833.9 (775.6–940.6)	0.05
*p*-value **(baseline *vs* follow-up)**	-	0.0009	0.08	-
**Relative change, % (baseline *vs* follow-up)**	−8.8 (-15.9–−1.6)	−11.7 (-18.8–−6.2)	−6.1 (-12.54–1.9)	0.02
**Mid-slice ADC, 10^−6^ mm^2^/s**				
**Baseline MRI**	842.7 (771.4–935.4)	817.1 (729.3–888.7)	865.5 (799.4–1010.0)	0.07
**Follow-up MRI**	802.4 (736.0–888.0)	739.8 (678.4–819.0)	795.0 (742.1–918.3)	0.02
*p*-value **(baseline *vs* follow-up)**	-	0.03	0.12	-
**Relative change, % (baseline *vs* follow-up)**	−3.3 (-15.0–6.0)	−9.5 (-13.6–−2.6)	−5.6 (-16.2–5.6)	0.79
**Small-volume ADC, 10^−6^ mm^2^/s**				
**Baseline MRI**	642.3 (588.8–723.6)	630.6 (580.4–694.2)	658.1 (599.6–757.3)	0.22
**Follow-up MRI**	596.3 (540.4–682.3)	580.3 (507.2–626.4)	640.6 (567.2–753.9)	0.004
*p*-value **(baseline *vs* follow-up)**	-	0.01	0.71	-
**Relative change, % (baseline *vs* follow-up)**	−7.4 (-15.0–0.5)	−9.5 (-15.5–−5.1)	−1.6 (-14.5–8.1)	0.008

ADC, apparent diffusion coefficient; *T*_2_WI, *T*_2_ weighted imaging.

The data are presented as median (interquartile range). The *p*-values were derived using the Mann–Whitney *U* test and are presented for intergroup comparisons between the absolute *T*_2_WI-derived measurements obtained from progressors and non-progressors, as well as between relative changes in the measurements derived from baseline and follow-up MRI scans

### Comparison of tumour measurements and ADC values in patients with and without radiological disease progression

When patients were regrouped based on the presence of radiological disease progression only, the trends in tumour measurements (Supplementary Material 1 **)** and ADC values (Supplementary Table 3) were similar compared to those reported above when the primary outcome was histopathological disease progression.

### Diagnostic performance of selected tumour measurements and ADC cut-offs

**Figure 3.**
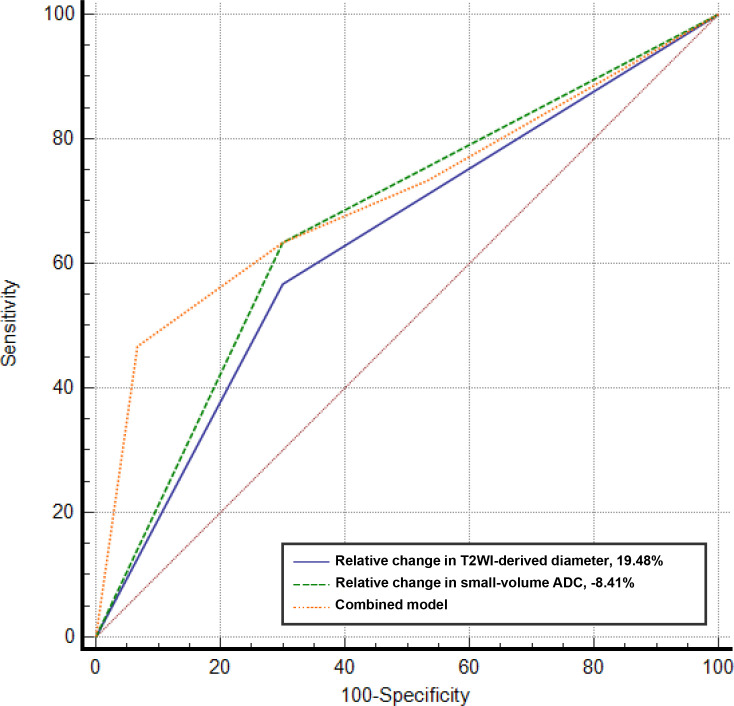
ROC curves for specific cut-offs of relative changes in *T*_2_WI-derived maximum tumour diameter and small-volume ADC values, as well as their combined model, used to detect histopathological prostate cancer progression in patients on active surveillance. ADC, apparent diffusion coefficient; ROC, receiver operating characteristic; *T*_2_WI, *T*_2_ weighted imaging.

**Figure 4.**
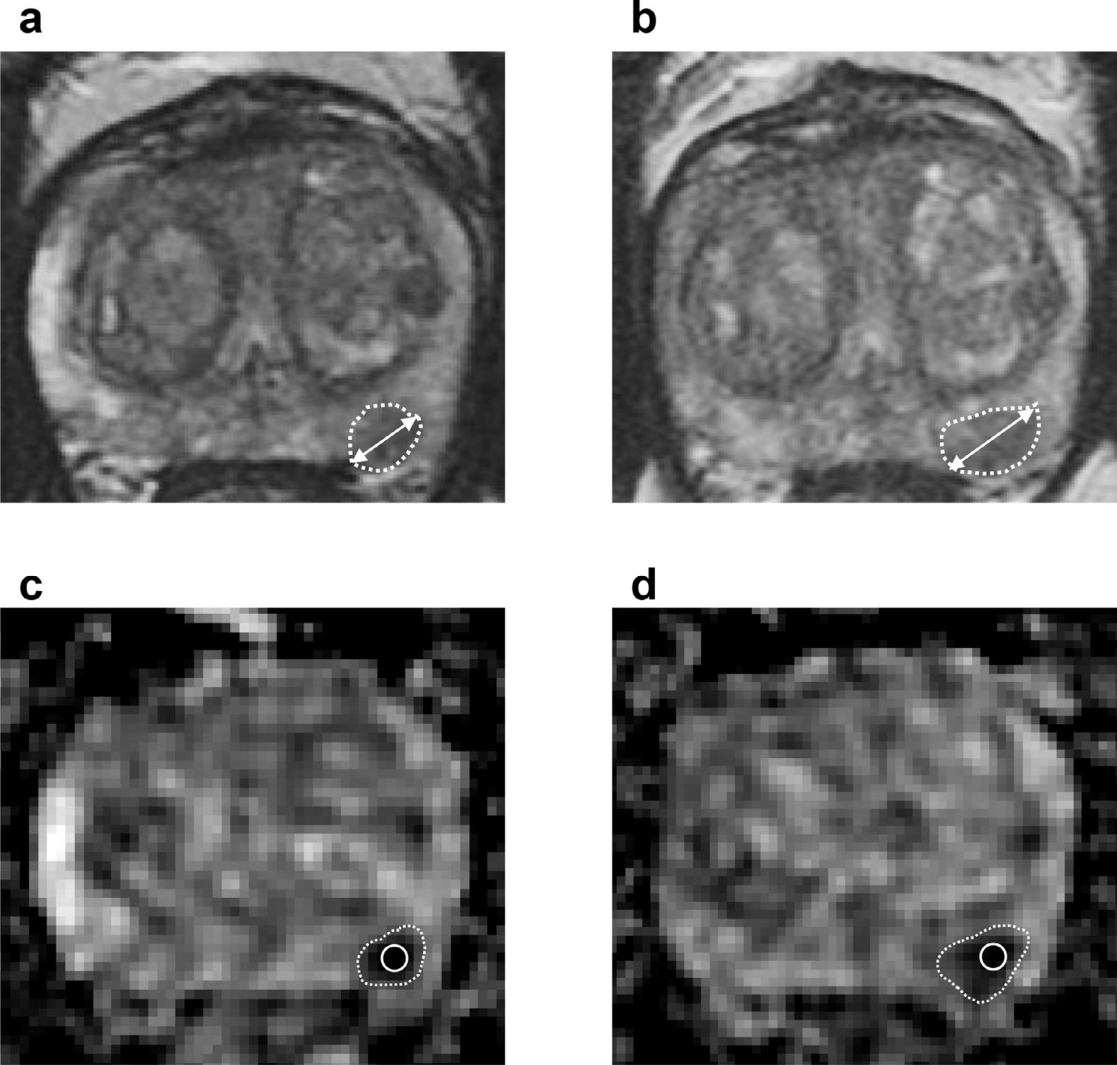
Axial *T*_2_WI (**A, B**) and ADC maps (**C, D**) obtained from a 60-year-old patient enrolled on active surveillance of an ISUP Grade 1 left posterior peripheral zone lesion. Baseline MRI (**A, C**) demonstrated the presence of a Likert 4 lesion measuring 9.4 mm in its maximum diameter (A, white double arrow) and having a small-volume ADC of 636 × 10^−6^ mm^2^/s (C, white circle). On a follow-up MRI scan performed at 24 months, the lesion was assigned a PRECISE 4 category and had a maximum diameter of 12.8 mm (+26.5%) (B, white double arrow) and a small-volume ADC of 566 × 10^−6^ mm^2^/s (−12.3%) (D, white circle). A subsequent biopsy confirmed histopathological progression of the outlined lesion to ISUP Grade 2, which prompted a switch to radical prostatectomy. ADC, apparent diffusion coefficient; *T*_2_WI, *T*_2_ weighted imaging.

**Figure 5.**
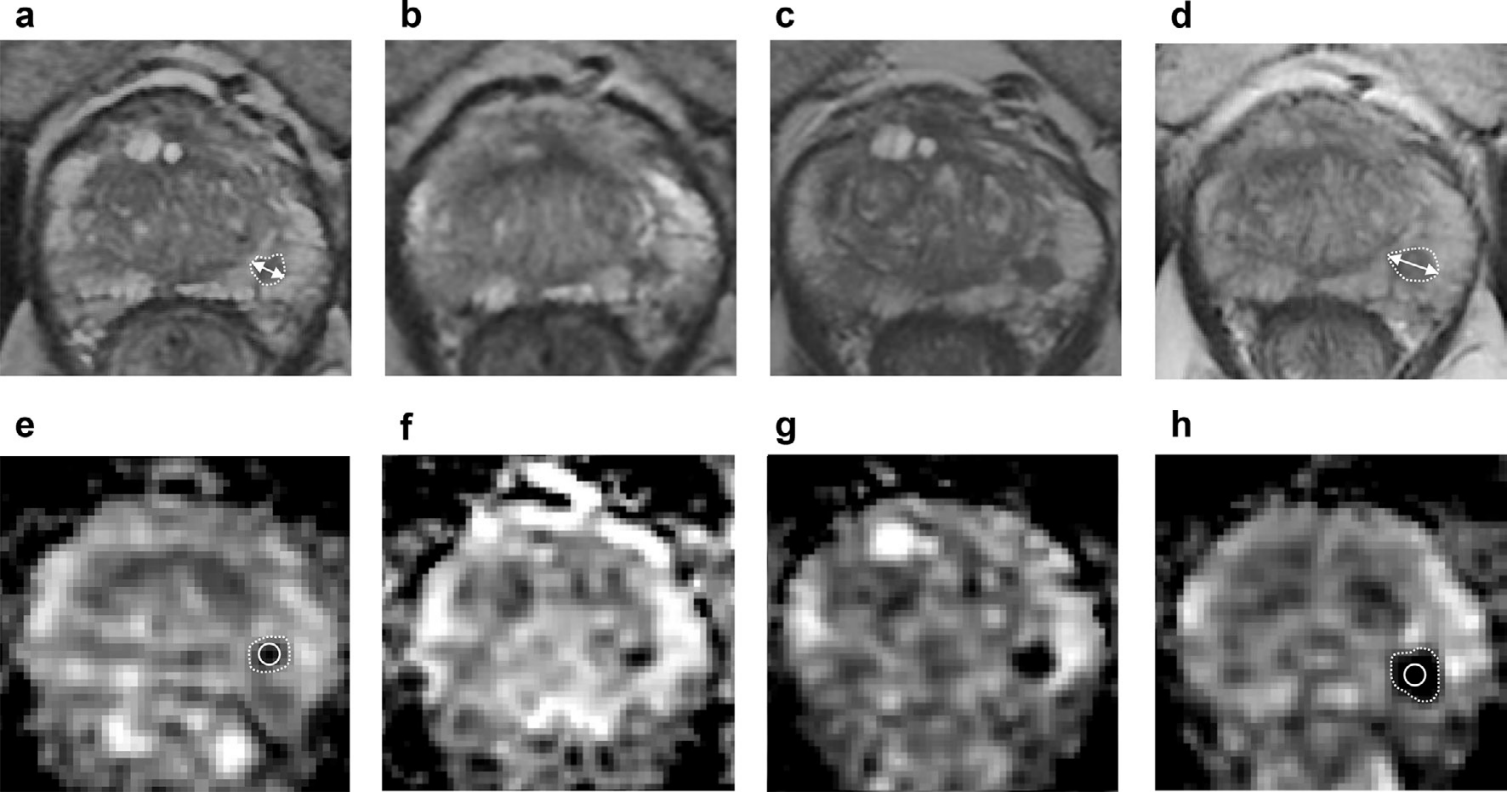
Axial *T*_2_WI (**A–D**) and ADC maps (**E–H**) obtained from a 69-year-old patient enrolled on active surveillance of an ISUP Grade 1 left posterior peripheral zone lesion. Baseline MRI (**A, E**) demonstrated a Likert 4 lesion with a maximum diameter of 5.5 mm (A, double arrow) and a small-volume ADC of 799 × 10^−6^ mm^2^/s (E, white circle). Follow-up scans were performed at 8 (**B, F**), 21 (**C, G**), 28 (not shown), and 40 (**D, H**) months. At 40 months (**D, H**), the lesion had a maximum diameter of 8.3 mm (+51% compared to baseline) (D, white double arrow) and a small-volume ADC of 589 × 10^−6^ mm^2^/s (−35.8% compared to baseline) (I, white circle). A repeat biopsy performed at 42 months post baseline MRI showed histopathological progression of the target lesion to ISUP Grade 5, which triggered a switch to androgen deprivation therapy followed by an external beam radiation therapy. Notably, the *T*_2_WI-derived maximum tumour diameter and small-volume ADC cut-offs proposed in the main text were first reached at 21 months post baseline MRI (**C, G**), being 6.6 mm (+20%) and 685.1 × 10^−6^ mm^2^/s (−17%), respectively, which could have been used to trigger repeat biopsy earlier. ADC, apparent diffusion coefficient; *T*_2_WI, *T*_2_ weighted imaging

AUCs for detecting histopathological disease progression based on relative changes in each of the *T*_2_WI-derived tumour measurements and ADC values are summarised in Table 4. As shown in Supplementary Material 1, *T*_2_WI-derived tumour measurements had significantly larger AUCs compared only to mid-slice ADC values (*p*-value range, 0.001–0.046). Although *T*_2_WI-derived 2D tumour surface area had the largest AUC (0.83) of all three tumour measurements, the DeLong test showed no significant difference between the three AUCs (*p*-value range, 0.140–0.526). Simultaneously, the AUC of mid-slice ADC (0.52) was significantly lower compared to those of whole-volume ADC (AUC = 0.67, *p* = 0.04) and small-volume ADC (AUC = 0.70, *p* = 0.003), with no difference observed between the AUCs of the latter two parameters (*p* = 0.72). Based on these results, and mirroring clinical practice, we chose *T*_2_WI-derived maximum tumour diameter and small-volume ADC as parameters to identify individual cut-offs that could be applied clinically. The selection of individual cut-offs (full list provided in Supplementary Material 1) for the final predictive modelling was based on prioritising specificity over sensitivity, considered more clinically relevant in order to reduce the need for repeat biopsies. Optimum results were achieved for a *T*_2_WI-derived maximum tumour diameter cut-off of 19.5% increase (specificity 73.4, sensitivity 56.7, AUC = 0.63) and a small-volume ROI-derived ADC reduction of −8.4% (specificity 70.0, sensitivity 63.3, AUC = 0.67). The combined model including both these values resulted in an AUC of 0.71 (Figure 3), which did not provide a significant improvement when compared to either individual parameter (*p* = 0.20 and *p* = 0.21). A separate model was also built for more clinically applicable cut-offs of 20% increase in *T*_2_WI-derived maximum tumour diameter and −10% reduction in small-volume ADC, with the resulting AUC being 0.67, which was similar to the aforementioned model (*p* = 0.29). Clinical examples using the proposed cut-offs are illustrated in Figures 4–5. As shown in Supplementary Material 1, the proposed cut-off of 20% increase in *T*_2_WI-derived maximum tumour diameter can also be supplemented by a minimum absolute increase in diameter to improve specificity, with a minimum size threshold of 3 mm likely to be optimal in this regard.

**Table 4.** AUC for detecting histopathological progression in patients on active surveillance for *T*_2_WI-derived tumour measurements and ADC values

Parameter	AUC	Standard error	95% confidence interval
*T*_2_WI-derived tumour volume	0.794	0.057	0.670 to 0.888
*T*_2_WI-derived 2D tumour surface area	0.828	0.052	0.708 to 0.913
*T*_2_WI-derived maximum tumour diameter	0.721	0.066	0.590 to 0.829
Whole-volume ADC	0.669	0.073	0.535 to 0.785
Mid-slice ADC	0.521	0.080	0.388 to 0.652
Small-volume ADC	0.698	0.072	0.566 to 0.810

ADC, apparent diffusion coefficient; AUC, areas under the ROC curves; 2D, two-dimensional; *T*_2_WI, *T*_2_ weighted imaging.

## Discussion

In this study, we demonstrate that follow-up changes in *T*_2_WI-derived tumour volume, 2D tumour surface area, and maximum tumour diameter, alongside whole-volume and small-volume ADC values were significantly different in AS patients with PCa histopathological progression compared to patients with stable disease. Specific cut-offs for *T*_2_WI-derived maximum tumour diameter (+20%) and small-volume ADC (−10%) values were proposed, which could be used in routine clinical practice to supplement subjective serial MRI assessment using PRECISE recommendations. These results pave the way for future prospective multicentre studies investigating the added value of the proposed cut-offs for improving the quality of AS programmes.

Here, we show that all three *T*_2_WI-derived tumour measurements demonstrated comparable diagnostic performance for predicting histopathological progression on AS, consistent with a previous study by Giganti et al.^20^ Clinically, evaluating serial changes in maximum tumour diameter closely mirrors the RECIST 1.1 criteria^30^ used in oncology to assess treatment outcomes. In our study, the median relative change in *T*_2_WI-derived maximum tumour diameter in progressors was 21.7%, which corresponded to the doubling of the median tumour volume in this group. These findings align with the RECIST 1.1 criteria for progressive disease, defined as a 20% increase in the sum of diameters of target lesions alongside a minimum of 5 mm absolute increase.^30^ 20% increase in *T*_2_WI diameter can be supplemented by a minimum of 3 mm absolute increase in tumour diameter, which corresponds to the 3 mm MRI slice thickness used in this study and may help mitigate the effect of interscan variability highlighted in previous studies.^31^ Moreover, this may help avoid errors in measurements due to partial volume or reproducibility at smaller lesion sizes, mirroring the incorporation of a minimum size increase of 5 mm in RECIST criteria. Using these cut-offs to supplement subjective serial MRI assessment could help improve the quality of MRI-guided AS programmes and further reduce the need for unnecessary biopsies while increasing the positive predictive value for histopathological disease progression. A previous study has shown similar relative increase in *T*_2_WI-derived maximum tumour diameter in AS patients with radiological disease progression,^20^ which is expected since the increase in size is an integral feature of PRECISE categories 4–5.^24^ In addition, our findings are consistent with the annual increases in tumour volumes (up to 23%) and diameters (up to 7%) reported by Giganti et al^31^ on different magnets using different scanning protocols, which supports the potential clinical applicability and reproducibility of our results. Interestingly, in our study, tumours in patients without histopathological and radiological disease progression also showed an increase in size over the median 4-year follow-up, which is expected given the natural tendency of tumours to grow. This further demonstrates the need of identifying specific cut-offs to differentiate “clinically significant” increases in tumour size. In line with our findings, Shoji et al^32^ showed that a 25% increase in TRUS-based maximum tumour diameter significantly increases the risk of PCa histopathological progression on AS. In addition, maximum tumour diameter has been proposed as a risk-stratification tool for patients eligible for AS,^33^ which supports the prospective use of this metric in the AS setting and lays the foundation for further multicentre investigations.

ADC values derived using three different approaches showed poorer diagnostic performance compared to *T*_2_WI-derived tumour measurements. This may reflect the known issues with repeatability and reproducibility of ADC in the prostate.^34–36^ In our study, all MRI examinations were performed on the same magnet with identical DWI acquisition parameters and selection of b-values for ADC map calculation in order to minimise variability, and performance of ADC is likely to reduce further if data from multiple MR systems with varying protocols are used. The observed decrease in tumour ADC values in progressors is in line with a previous report^19^ where radiological progression was the primary clinical outcome. However, it should be noted that non-progressors also demonstrated an overall reduction in tumour-derived ADC values, similar to the whole-gland ADC measurements reported previously by Morgan et al.^21^ The small-volume ROI technique used in this study is of relevance as this directly mirrors clinical practice, and has been used extensively in other studies measuring prostate tumour-derived ADC.^37–39^ The promise shown by ADC for differentiating indolent *vs* clinically significant PCa warrants further work addressing the intrinsic limitations of ADC to maximise its clinical reliability, possibly with the incorporation of ADC ratios.^20,40^ However, the calculation of the latter in routine clinical practice has to be justified given the increasing pressure on imaging services and the need to reduce the reporting time.^25^

This hypothesis-generating study has some limitations. The sample size is relatively small, which was dictated by stringent inclusion criteria aimed at reducing confounding factors that may compromise robust comparisons and ensuring histopathologically confirmed disease progression as a gold standard. While we also showed similar in results in tumour diameters and ADC values when comparing radiological progressors and non-progressors, histopathology offers a more reliable reference standard compared to MRI alone. Importantly, this study excluded patients with MR-invisible lesions, who comprise nearly half of the AS population^23,41^ and to whom the proposed cut-offs are not applicable by definition. However, as evidenced by previous studies,^23,42^ the presence of MR-visible lesions presents a significant risk factor for PCa progression, which warrants closer surveillance of this patient subgroup. As highlighted, all scans analysed as part of this study were performed on the same clinical MRI system with an identical acquisition protocol, which may limit the generalisability of our ADC findings given the known low intra- and intersystem reproducibility and repeatability of ADC measurements. Future work should be aimed at validating these results in multicentre studies using data obtained from different scanners and with different protocols. Moreover, gaining access to larger cohorts will help evaluate the utility of the proposed cut-offs to predict histopathological progression between risk groups rather than individual grade groups, which may improve patient survival.^43^ Finally, combining the proposed cut-offs with other quantitative MRI features such as those derived from radiomics,^44,45^ alongside standard clinical biomarkers of disease progression, *e.g.* PSA and PSA density, may further improve their diagnostic performance and help objectivise serial MRI assessment in AS. The introduction of more complex predictive models may also help increase the applicability of the proposed cut-offs to individual cases, which in the present form may be limited given considerable interquartile and total range overlaps between progressors and non-progressors reported in this study. The reported overlaps may, however, provide a quantitative explanation for the overall low reliability of using serial MRI assessment alone for excluding PCa progression on AS.^9,10^

In conclusion, we show that patients with histopathological PCa progression on AS demonstrate a significant increase in *T*_2_WI-derived tumour measurements and ADC values. Relative changes in *T*_2_WI-derived tumour diameter and small-volume ADC values can be objectively evaluated in routine clinical practice, and their cut-off values of +20% and −10%, respectively, can be validated as part of multicentre studies and used to supplement the subjective PRECISE image assessment criteria.

## References

[1] Heidenreich A, Bellmunt J, Bolla M, Joniau S, Mason M, Matveev V, et al . EAU guidelines on prostate cancer. Part 1: screening, diagnosis, and treatment of clinically localised disease. Eur Urol 2011; 59: 61–71. doi: 10.1016/j.eururo.2010.10.039 21056534

[2] Mottet N, Bellmunt J, Bolla M, Briers E, Cumberbatch MG, De Santis M, et al . EAU-ESTRO-SIOG guidelines on prostate cancer. Part 1: screening, diagnosis, and local treatment with curative intent. Eur Urol 2017; 71: 618–29. doi: 10.1016/j.eururo.2016.08.003 27568654

[3] Negoita S, Feuer EJ, Mariotto A, Cronin KA, Petkov VI, Hussey SK, et al . Annual report to the nation on the status of cancer, part II: recent changes in prostate cancer trends and disease characteristics. Cancer 2018; 124: 2801–14. doi: 10.1002/cncr.31549 29786851 PMC6005761

[4] Results of the NPCA prospective audit in England and Wales for men diagnosed from 1 national prostate cancer audit seventh year annual Report-Results of the NPCA prospective audit in England and Wales for men diagnosed. 2020; 1.

[5] Van Hemelrijck M, Ji X, Helleman J, Roobol MJ, van der Linden W, Nieboer D, et al . Reasons for discontinuing active surveillance: assessment of 21 centres in 12 countries in the Movember GAP3 Consortium. Eur Urol 2019; 75: 523–31. doi: 10.1016/j.eururo.2018.10.025 30385049 PMC8542419

[6] Loeb S, Vellekoop A, Ahmed HU, Catto J, Emberton M, Nam R . Systematic review of complications of prostate biopsy. Eur Urol 2013;. 10.1016/j.eururo.2013.05.049 23787356

[7] Bokhorst LP, Alberts AR, Rannikko A, Valdagni R, Pickles T, Kakehi Y, et al . Compliance rates with the prostate cancer research International active surveillance (PRIAS) protocol and disease reclassification in Noncompliers. Eur Urol 2015; 68: 814–21. doi: 10.1016/j.eururo.2015.06.012 26138043

[8] Giganti F, Kirkham A, Allen C, Punwani S, Orczyk C, Emberton M, et al . Update on multiparametric prostate MRI during active surveillance: current and future trends and role of the precise recommendations. AJR Am J Roentgenol 2021; 216: 943–51. doi: 10.2214/AJR.20.23985 32755219

[9] Rajwa P, Pradere B, Quhal F, Mori K, Laukhtina E, Huebner NA, et al . Reliability of serial prostate magnetic resonance imaging to detect prostate cancer progression during active surveillance: a systematic review and meta-analysis. Eur Urol 2021;18 May 2021. doi: 10.1016/j.eururo.2021.05.001 34020828

[10] Hettiarachchi D, Geraghty R, Rice P, Sachdeva A, Nambiar A, Johnson M, et al . Can the Use of Serial Multiparametric Magnetic Resonance Imaging During Active Surveillance of Prostate Cancer Avoid the Need for Prostate Biopsies?-A Systematic Diagnostic Test Accuracy Review. Eur Urol Oncol 2021; 4: 426–36. doi: 10.1016/j.euo.2020.09.002 32972894

[11] Ploussard G, Renard-Penna R . Mri-Guided active surveillance in prostate cancer: not yet ready for practice. Nat Rev Urol 2021; 18: 77–8. doi: 10.1038/s41585-020-00416-2 33311651

[12] Thurtle D, Barrett T, Thankappan-Nair V, Koo B, Warren A, Kastner C, et al . Progression and treatment rates using an active surveillance protocol incorporating image-guided baseline biopsies and multiparametric magnetic resonance imaging monitoring for men with favourable-risk prostate cancer. BJU Int 2018; 122: 59–65. doi: 10.1111/bju.14166 29438586

[13] de Rooij M, Israël B, Tummers M, Ahmed HU, Barrett T, Giganti F, et al . ESUR/ESUI consensus statements on multi-parametric MRI for the detection of clinically significant prostate cancer: quality requirements for image acquisition, interpretation and radiologists' training. Eur Radiol 2020; 30: 5404–16. doi: 10.1007/s00330-020-06929-z 32424596 PMC7476997

[14] de Rooij M, Israël B, Barrett T, Giganti F, Padhani AR, Panebianco V, et al . Focus on the quality of prostate multiparametric magnetic resonance imaging: synopsis of the ESUR/ESUI recommendations on quality assessment and interpretation of images and radiologists' training. Eur Urol 2020; 78: 483–5. doi: 10.1016/j.eururo.2020.06.023 32591100

[15] Gaziev G, Wadhwa K, Barrett T, Koo BC, Gallagher FA, Serrao E, et al . Defining the learning curve for multiparametric magnetic resonance imaging (MRI) of the prostate using MRI-transrectal ultrasonography (TRUS) fusion-guided transperineal prostate biopsies as a validation tool. BJU Int 2016; 117: 80–6. doi: 10.1111/bju.12892 25099182

[16] Rosenkrantz AB, Ayoola A, Hoffman D, Khasgiwala A, Prabhu V, Smereka P, et al . The learning curve in prostate MRI interpretation: Self-directed learning versus continual reader feedback. AJR Am J Roentgenol 2017; 208: W92, ––100 p.. doi: 10.2214/AJR.16.16876 28026201

[17] Moore CM, Giganti F, Albertsen P, Allen C, Bangma C, Briganti A, et al . Reporting magnetic resonance imaging in men on active surveillance for prostate cancer: the precise Recommendations-A report of a European school of oncology Task force. Eur Urol 2017; 71: 648–55. doi: 10.1016/j.eururo.2016.06.011 27349615

[18] Padhani AR, Rouvière O, Schoots IG . Magnetic resonance imaging for tailoring the need to biopsy during follow-up for men on active surveillance for prostate cancer. Eur Urol 2021;27 May 2021. doi: 10.1016/j.eururo.2021.05.024 34053779

[19] Giganti F, Pecoraro M, Fierro D, Campa R, Del Giudice F, Punwani S, et al . Dwi and precise criteria in men on active surveillance for prostate cancer: a multicentre preliminary experience of different ADC calculations. Magn Reson Imaging 2020; 67: 50–8. doi: 10.1016/j.mri.2019.12.007 31899283

[20] Giganti F, Stavrinides V, Stabile A, Osinibi E, Orczyk C, Radtke JP, et al . Prostate cancer measurements on serial MRI during active surveillance: it's time to be precise. Br J Radiol 2020; 93: 20200819. doi: 10.1259/bjr.20200819 32955923 PMC7716009

[21] Morgan VA, Riches SF, Thomas K, Vanas N, Parker C, Giles S, et al . Diffusion-Weighted magnetic resonance imaging for monitoring prostate cancer progression in patients managed by active surveillance. Br J Radiol 2011; 84: 31–7. doi: 10.1259/bjr/14556365 21172965 PMC3473800

[22] Morgan VA, Parker C, MacDonald A, Thomas K, deSouza NM . Monitoring tumor volume in patients with prostate cancer undergoing active surveillance: is MRI apparent diffusion coefficient indicative of tumor growth? AJR Am J Roentgenol 2017; 209: 620–8. doi: 10.2214/AJR.17.17790 28609110

[23] Caglic I, Sushentsev N, Gnanapragasam VJ, Sala E, Shaida N, Koo BC, et al . MRI-derived precise scores for predicting pathologically-confirmed radiological progression in prostate cancer patients on active surveillance. Eur Radiol 2021; 31: 1–10. doi: 10.1007/s00330-020-07336-0 33196886 PMC8043947

[24] Giganti F, Pecoraro M, Stavrinides V, Stabile A, Cipollari S, Sciarra A, et al . Interobserver reproducibility of the precise scoring system for prostate MRI on active surveillance: results from a two-centre pilot study. Eur Radiol 2020; 30: 2082–90. doi: 10.1007/s00330-019-06557-2 31844959 PMC7062656

[25] Sushentsev N, Caglic I, Sala E, Shaida N, Slough RA, Carmo B, et al . The effect of capped biparametric magnetic resonance imaging slots on Weekly prostate cancer imaging workload. Br J Radiol 2020; 93: 20190929. doi: 10.1259/bjr.20190929 31971823 PMC7362922

[26] Yushkevich PA, Piven J, Hazlett HC, Smith RG, Ho S, Gee JC, et al . User-guided 3D active contour segmentation of anatomical structures: significantly improved efficiency and reliability. Neuroimage 2006; 31: 1116–28. doi: 10.1016/j.neuroimage.2006.01.015 16545965

[27] Turkbey B, Rosenkrantz AB, Haider MA, Padhani AR, Villeirs G, Macura KJ, et al . Prostate imaging reporting and data system version 2.1: 2019 update of prostate imaging reporting and data system version 2. Eur Urol 2019; 76: 340–51. doi: 10.1016/j.eururo.2019.02.033 30898406

[28] Barrett T, Padhani AR, Patel A, Ahmed HU, Allen C, Bardgett H, et al . Certification in reporting multiparametric magnetic resonance imaging of the prostate: recommendations of a UK consensus meeting. BJU Int 2021; 127: 304-306. doi: 10.1111/bju.15285 33113258

[29] Barrett T, Rajesh A, Rosenkrantz AB, Choyke PL, Turkbey B . PI-RADS version 2.1: one small step for prostate MRI. Clin Radiol 2019; 74: 841–52. doi: 10.1016/j.crad.2019.05.019 31239107

[30] Eisenhauer EA, Therasse P, Bogaerts J, Schwartz LH, Sargent D, Ford R, et al . New response evaluation criteria in solid tumours: revised RECIST guideline (version 1.1. Eur J Cancer 2009; 45: 228–47. doi: 10.1016/j.ejca.2008.10.026 19097774

[31] Giganti F, Allen C, Stavrinides V, Stabile A, Haider A, Freeman A . ] Tumour growth rates of prostate cancer during active surveillance: is there a difference between MRI-visible low and intermediate-risk disease? et al.. Available from: 10.1259/BJR.20210321. PMC8978245 34233491

[32] Shoji S, Ukimura O, de Castro Abreu AL, Marien A, Matsugasumi T, Bahn D, et al . Image-Based monitoring of targeted biopsy-proven prostate cancer on active surveillance: 11-year experience. World J Urol 2016; 34: 221–7. doi: 10.1007/s00345-015-1619-z 26093647 PMC9084468

[33] Lee DH, Koo KC, Lee SH, Rha KH, Choi YD, Hong SJ, et al . Tumor lesion diameter on diffusion weighted magnetic resonance imaging could help predict insignificant prostate cancer in patients eligible for active surveillance: preliminary analysis. J Urol 2013; 190: 1213–7. doi: 10.1016/j.juro.2013.03.127 23727188

[34] Barrett T, Lawrence EM, Priest AN, Warren AY, Gnanapragasam VJ, Gallagher FA, et al . Repeatability of diffusion-weighted MRI of the prostate using whole lesion ADC values, skew and histogram analysis. Eur J Radiol 2019; 110: 22–9. doi: 10.1016/j.ejrad.2018.11.014 30599864

[35] Tamada T, Huang C, Ream JM, Taffel M, Taneja SS, Rosenkrantz AB . Apparent diffusion coefficient values of prostate cancer: comparison of 2D and 3D ROIs. AJR Am J Roentgenol 2018; 210: 113–7. doi: 10.2214/AJR.17.18495 29045185

[36] Fedorov A, Vangel MG, Tempany CM, Fennessy FM . Multiparametric magnetic resonance imaging of the prostate: repeatability of volume and apparent diffusion coefficient quantification. Invest Radiol 2017; 52: 538–46. doi: 10.1097/RLI.0000000000000382 28463931 PMC5544576

[37] De Cobelli F, Ravelli S, Esposito A, Giganti F, Gallina A, Montorsi F, et al . Apparent diffusion coefficient value and ratio as noninvasive potential biomarkers to predict prostate cancer grading: comparison with prostate biopsy and radical prostatectomy specimen. AJR Am J Roentgenol 2015; 204: 550–7. doi: 10.2214/AJR.14.13146 25714284

[38] Jyoti R, Jain TP, Haxhimolla H, Liddell H, Barrett SE . Correlation of apparent diffusion coefficient ratio on 3.0 T MRI with prostate cancer Gleason score. Eur J Radiol Open 2018; 5: 58–63. doi: 10.1016/j.ejro.2018.03.002 29687050 PMC5910169

[39] Boesen L, Chabanova E, Løgager V, Balslev I, Thomsen HS . Apparent diffusion coefficient ratio correlates significantly with prostate cancer Gleason score at final pathology. J Magn Reson Imaging 2015; 42: 446–53. doi: 10.1002/jmri.24801 25408104

[40] Barrett T, Priest AN, Lawrence EM, Goldman DA, Warren AY, Gnanapragasam VJ, et al . Ratio of tumor to normal prostate tissue apparent diffusion coefficient as a method for quantifying DWI of the prostate. AJR Am J Roentgenol 2015; 205: W585–93. doi: 10.2214/AJR.15.14338 26587948

[41] Stavrinides V, Giganti F, Trock B, Punwani S, Allen C, Kirkham A, et al . Five-Year outcomes of magnetic resonance Imaging–based active surveillance for prostate cancer: a large cohort study. Eur Urol 2020; 78: 443–51. doi: 10.1016/j.eururo.2020.03.035 32360049 PMC7443696

[42] Stavrinides V, Giganti F, Trock B, Punwani S, Allen C, Kirkham A, et al . Five-Year outcomes of magnetic resonance imaging-based active surveillance for prostate cancer: a large cohort study. Eur Urol 2020; 78: 443–51. doi: 10.1016/j.eururo.2020.03.035 32360049 PMC7443696

[43] Gnanapragasam VJ, Barrett T, Thankapannair V, Thurtle D, Rubio-Briones J, Domínguez-Escrig J, et al . Using prognosis to guide inclusion criteria, define standardised endpoints and stratify follow-up in active surveillance for prostate cancer. BJU Int 2019; 124: 758–67. doi: 10.1111/bju.14800 31063245

[44] Sushentsev N, Rundo L, Blyuss O, Gnanapragasam VJ, Sala E, Barrett T . MRI-derived radiomics model for baseline prediction of prostate cancer progression on active surveillance. Sci Rep 2021; 11: 12917. doi: 10.1038/s41598-021-92341-6 34155265 PMC8217549

[45] Sushentsev N, Rundo L, Blyuss O, Nazarenko T, Suvorov A, Gnanapragasam VJ, et al . Comparative performance of MRI-derived precise scores and delta-radiomics models for the prediction of prostate cancer progression in patients on active surveillance. Eur Radiol 2021; 2021: 1–10. doi: 10.1007/s00330-021-08151-x 34255161 PMC8660717

