# Supplementary material for: Serial changes in tumour measurements and apparent diffusion coefficients in prostate cancer patients on active surveillance with and without histopathological progression
Source: Br J Radiol. 2021 Sep 19;95(1131):20210842. doi: 10.1259/bjr.20210842 (PMC8978242; doi:10.1259/bjr.20210842)
Supplement: Supplementary Material 1. [file bjr.20210842.suppl-01.pdf]

Serial changes in tumour measurements and apparent diffusion coefficients in prostate cancer patients on active surveillance with and without histopathological progression

Supplementary Materials.

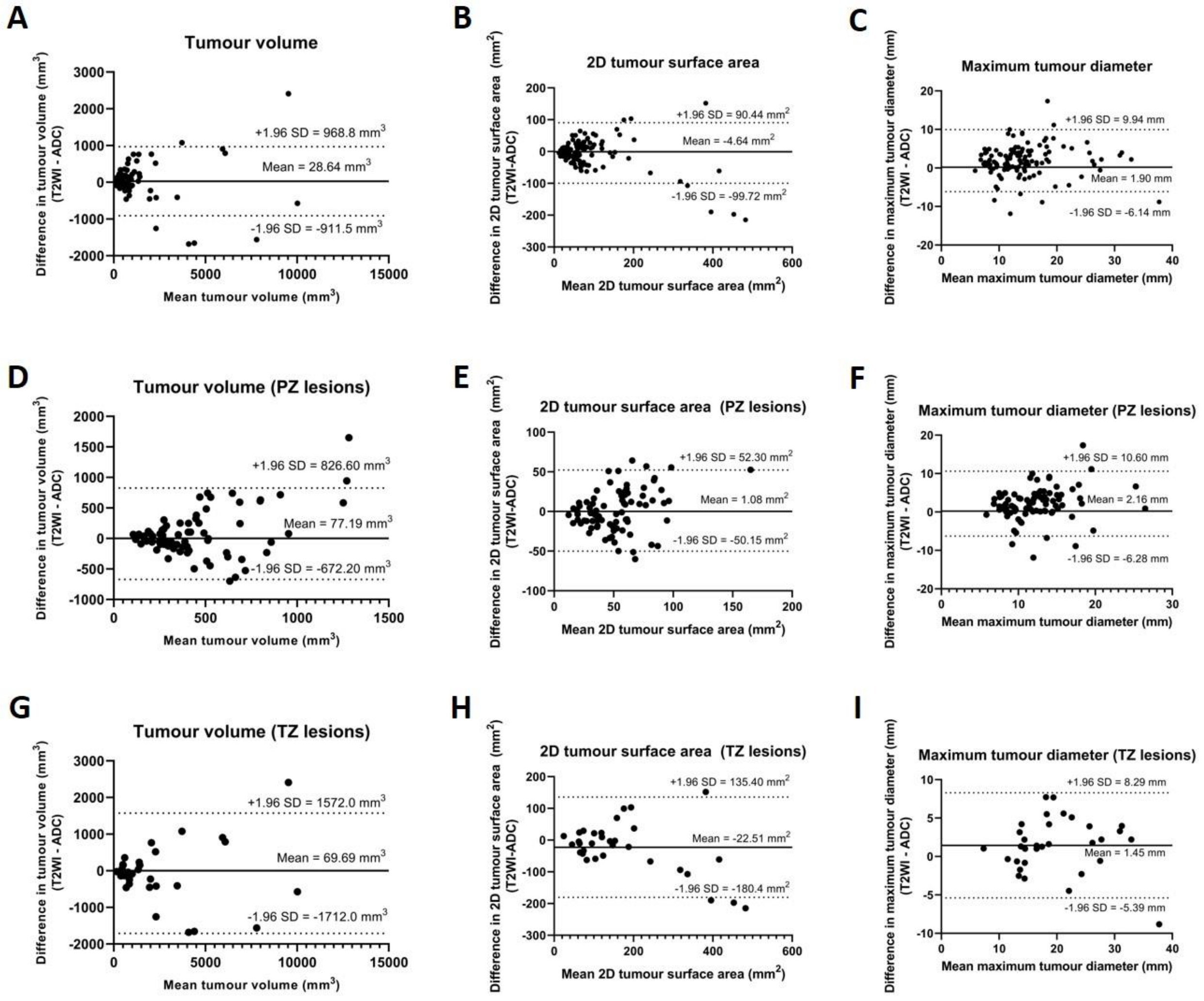

Supplementary Figure 1. Bland–Altman plots comparing T2WI- and ADC-derived tumour volumes, 2D tumour surface areas, and maximum tumour diameters derived from all lesions (A-C), PZ lesions only (D-F), and TZ lesions only (G-I). Dotted lines represent upper and lower 95% limits of agreement, and bold lines represent the mean biases with appropriate captions included. ADC = apparent diffusion coefficient, PZ = peripheral zone, T2WI = T_2-weighted imaging, TZ = transition zone.

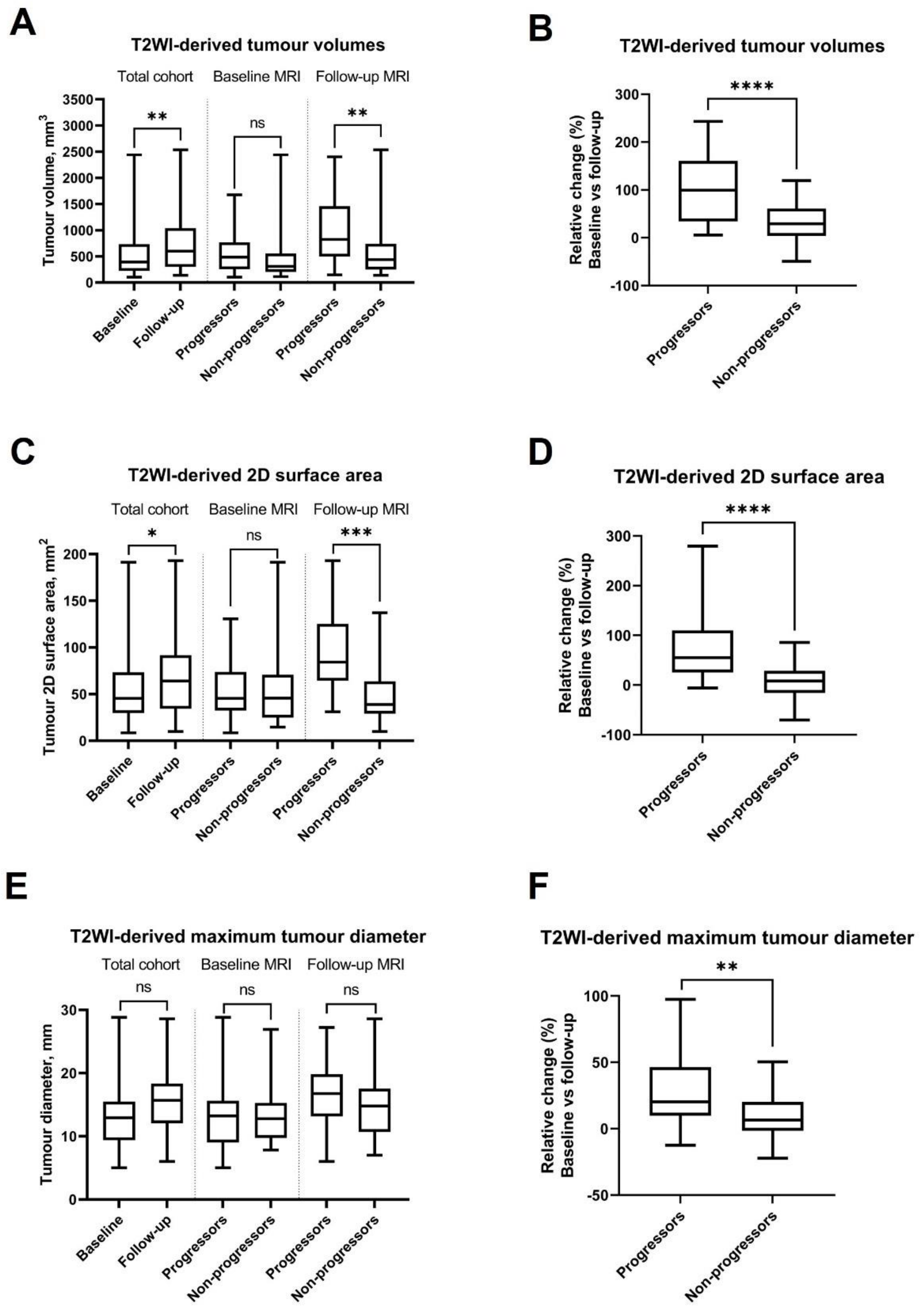

Supplementary Figure 2. Box-and-whisker plots comparing absolute (A, C, E) and relative (B, D, F) changes in T2WI-derived tumour volumes (A, B), 2D surface areas (C, D), and maximum tumour diameters (E, F) at baseline and follow-up MRI studies, as well as between progressors and non-progressors at the two time points. Top and bottom of boxes represent 25th and 75th percentiles of data, respectively; line in boxes represents the median value and bars represent minimum and maximum values. The figure supplements **Table 2** of the main text.

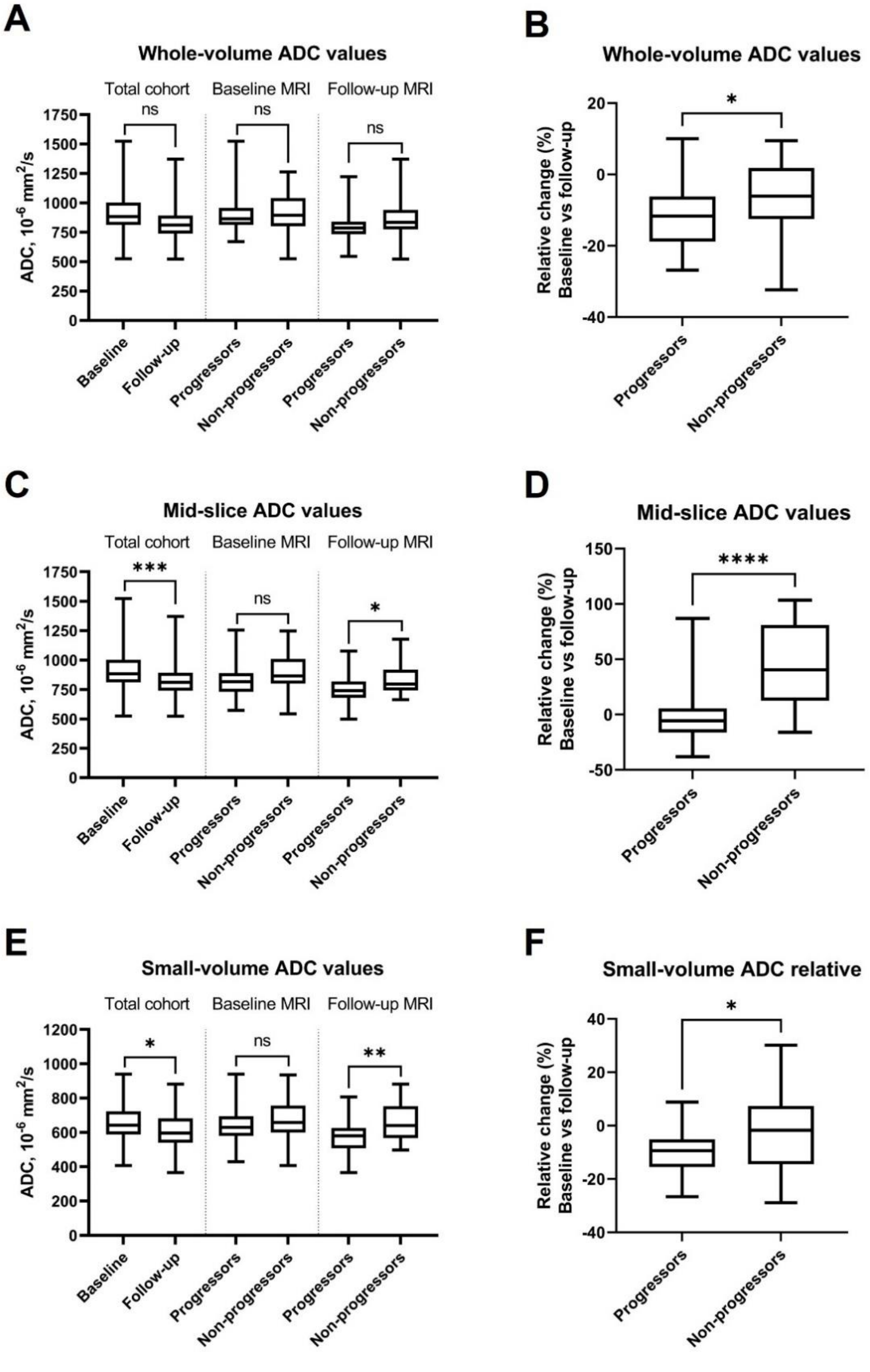

Supplementary Figure 3. Box-and-whisker plots comparing absolute (A, C, E) and relative (B, D, F) changes in whole-volume (A, B), mid-slice (C, D), and small-volume (E, F) ADC values at baseline and follow-up MRI studies, as well as between progressors and non-progressors at the two time points. Top and bottom of boxes represent 25th and 75th percentiles of data, respectively; line in boxes represents the median value and bars represent minimum and maximum values. The figure supplements **Table 3** of the main text.

Correlation pair	Spearman's ρ	95% confidence interval of Spearman's ρ	p-value
T2WI-derived tumour volume and ADC-derived tumour volume (all lesions)	0.92	0.88 to 0.94	<0.0001
T2WI-derived 2D tumour surface area and ADC-derived 2D tumour surface area (all lesions)	0.75	0.66 to 0.82	<0.0001
T2WI-derived maximum tumour diameter and ADC-derived maximum tumour diameter (all lesions)	0.67	0.55 to 0.76	<0.0001
T2WI-derived tumour volume and ADC-derived tumour volume (PZ lesions)	0.45	0.23 to 0.62	<0.0001
T2WI-derived 2D tumour surface area and ADC-derived 2D tumour surface area (PZ lesions)	0.56	0.39 to 0.70	<0.0001
T2WI-derived maximum tumour diameter and ADC-derived maximum tumour diameter (PZ lesions)	0.50	0.31 to 0.65	<0.0001
T2WI-derived tumour volume and ADC-derived tumour volume (TZ lesions)	0.95	0.89 to 0.98	<0.0001
T2WI-derived 2D tumour surface area and ADC-derived 2D tumour surface area (TZ lesions)	0.88	0.76 to 0.94	<0.0001
T2WI-derived maximum tumour diameter and ADC-derived maximum tumour diameter (TZ lesions)	0.86	0.73 to 0.93	<0.0001

Supplementary Table 1. Outputs of the Spearman's correlation analysis underlying Fig. 3 of the main text.

Parameter	Radiological progression (n = 27)	Radiologically stable disease (n = 33)	p-value
**T2WI-derived tumour volume (mm^3)**			
**Baseline MRI**	477.2 (288.7-827.6)	333.9 (202.5-785.1)	0.32
**Follow-up MRI**	835.4 (526.6-1500.0)	471.5 (288.5-942.3)	0.06
**p-value (baseline vs follow-up)**	0.009	0.12	-
**Relative change (%) (baseline vs follow-up)**	69.1 (28.0-154.0)	50.9 (8.5-81.1)	0.02
**T2WI-derived 2D tumour surface area (mm^2)**			
**Baseline MRI**	56.2 (33.2-74.3)	46.2 (25.7-100.5)	0.62
**Follow-up MRI**	84.3 (57.5-126.1)	49.1 (32.3-97.1)	0.03
**p-value (baseline vs follow-up)**	0.008	0.54	-
**Relative change (%) (baseline vs follow-up)**	51.3 (11.6-87.7)	17.8 (-6.4-45.3)	0.02
**T2WI-derived maximum tumour diameter (mm)**			
**Baseline MRI**	13.1 (9.0-15.3)	12.9 (10.0-17.0)	0.514
**Follow-up MRI**	17.0 (13.6-20.0)	15.0 (11.3-18.2)	0.210
**p-value (baseline vs follow-up)**	0.003	0.17	-
**Relative change (%) (baseline vs follow-up)**	23.1 (8.6-48.4)	10.4 (0.3-23.4)	0.02

Supplementary Table 2. T2WI-derived tumour measurements obtained from baseline and latest available follow-up MRI scans in patients on active surveillance who showed radiological progression of the disease (PRECISE 4-5) versus men whose disease remained radiologically stable (PRECISE 1-3). The data are presented as median (interquartile range). The p-values were derived using the Mann-Whitney U test and are presented for intergroup comparisons between the absolute T2WI-derived measurements obtained from progressors and non-progressors, baseline and follow-up scans in patients from the same groups, as well as between relative changes in the measurements derived from baseline and follow-up MRI scans.

Parameter	Radiological progression (n = 27)	Radiologically stable disease (n = 33)	p-value
**Whole-volume ADC, 10^{-6} mm²/s**			
**Baseline MRI**	417.7 (221.6-845.1)	356.2 (194.5-864.7)	0.70
**Follow-up MRI**	689.4 (458.5-1325.0)	461.7 (246.3-928.4)	0.11
**p-value (baseline vs follow-up)**	0.03	0.44	-
**Relative change (%) (baseline vs follow-up)**	75.5 (23.8-189.8)	28.5 (-5.6-70.6)	0.008
**Mid-slice ADC, 10^{-6} mm²/s**			
**Baseline MRI**	58.9 (33.5-80.0)	49.1 (32.3-82.1)	0.583
**Follow-up MRI**	74.4 (49.3-117.8)	58.6 (40.1-95.7)	0.121
**p-value (baseline vs follow-up)**	0.05	0.47	-
**Relative change (%) (baseline vs follow-up)**	39.4 (13.0-79.3)	22.3 (-16.5-49.5)	0.05
**Small-volume ADC, 10^{-6} mm²/s**			
**Baseline MRI**	11.6 (8.6-13.4)	10.7 (7.9-16.8)	0.988
**Follow-up MRI**	13.9 (10.3-16.6)	12.2 (9.3-15.1)	0.188
**p-value (baseline vs follow-up)**	0.02	0.45	-
**Relative change (%) (baseline vs follow-up)**	23.2 (14.6-36.3)	6.5 (-8.9-26.3)	0.004

Supplementary Table 3. Tumour ADC values derived from baseline and follow-up MRI scans in active surveillance patients who showed radiological progression of the disease (PRECISE 4-5) and whose disease remained radiologically stable (PRECISE 1-3). The data are presented as median (interquartile range). The p-values were derived using the Mann-Whitney U test and are presented for intergroup comparisons between the absolute T2WI-derived measurements obtained from progressors and non-progressors, baseline and follow-up scans in patients from the same groups, as well as between relative changes in the measurements derived from baseline and follow-up MRI scans.

Parameter	T2WI-derived tumour volume	T2WI-derived 2D tumour surface area	T2WI-derived maximum tumour diameter
**Whole-volume ADC**	0.169	0.061	0.547
**Mid-slice ADC**	0.006	0.001	0.046
**Small-volume ADC**	0.299	0.121	0.808

Supplementary Table 4. Outputs of the DeLong test comparing areas under the ROC curves between T2WI-derived tumour measurements and ADC values.

Cut-off value	Sensitivity	95% CI	Specificity	95% CI	PPV	NPV
≥-22.2	100.00	88.4 – 100.0	0.00	0.0 – 11.6	50.0	-
>-12.75	100.00	88.4 – 100.0	6.67	0.8 – 22.1	51.7	100.0
>-12.5	96.67	82.8 – 99.9	6.67	0.8 – 22.1	50.9	66.7
>-1.32	96.67	82.8 – 99.9	26.67	12.3 – 45.9	56.9	88.9
>-0.98	93.33	77.9 – 99.2	26.67	12.3 – 45.9	56.0	80.0
>1.58	93.33	77.9 – 99.2	33.33	17.3 – 52.8	58.3	83.3
>1.90	90.00	73.5 – 97.9	33.33	17.3 – 52.8	57.4	76.9
>4.35	90.00	73.5 – 97.9	43.33	25.5 – 62.6	61.4	81.2
>6.20	83.33	65.3 – 94.4	43.33	25.5 – 62.6	59.5	72.2
>7.59	83.33	65.3 – 94.4	56.67	37.4 – 74.5	65.8	77.3
>10.39	73.33	54.1 – 87.7	56.67	37.4 – 74.5	62.9	68.0
>10.72	73.33	54.1 – 87.7	60.00	40.6 – 77.3	64.7	69.2
>11.07	70.00	50.6 – 85.3	60.00	40.6 – 77.3	63.6	66.7
>12.50	70.00	50.6 – 85.3	63.33	43.9 – 80.1	65.6	67.9
>13.03	66.67	47.2 – 82.7	63.33	43.9 – 80.1	64.5	65.5
>17.02	66.67	47.2 – 82.7	66.67	47.2 – 82.7	66.7	66.7
>18.38	56.67	37.4 – 74.5	66.67	47.2 – 82.7	63.0	60.6
>19.48	56.67	37.4 – 74.5	73.33	54.1 – 87.7	68.0	62.9
>20.35	50.00	31.3 – 68.7	73.33	54.1 – 87.7	65.2	59.5
>20.96	50.00	31.3 – 68.7	76.67	57.7 – 90.1	68.2	60.5
>23.09	46.67	28.3 – 65.7	76.67	57.7 – 90.1	66.7	59.0
>25.77	46.67	28.3 – 65.7	80.00	61.4 – 92.3	70.0	60.0
>28.64	40.00	22.7 – 59.4	80.00	61.4 – 92.3	66.7	57.1
>29.45	40.00	22.7 – 59.4	83.33	65.3 – 94.4	70.6	58.1
>33.31	36.67	19.9 – 56.1	83.33	65.3 – 94.4	68.8	56.8
>35.01	36.67	19.9 – 56.1	90.00	73.5 – 97.9	78.6	58.7
>37.52	33.33	17.3 – 52.8	90.00	73.5 – 97.9	76.9	57.4
>37.89	33.33	17.3 – 52.8	93.33	77.9 – 99.2	83.3	58.3
>48.44	23.33	9.9 – 42.3	93.33	77.9 – 99.2	77.8	54.9
>50.33	23.33	9.9 – 42.3	96.67	82.8 – 99.9	87.5	55.8
>97.49	3.33	0.08 – 17.2	96.67	82.8 – 99.9	50.0	50.0
>103.56	3.33	0.08 – 17.2	100.00	88.4 – 100.0	100.0	50.8
>188.30	0.00	0.0 – 11.6	100.00	88.4 – 100.0	-	50.0

Supplementary Table 5. Summary performance characteristics of individual T2WI-derived maximum tumour diameter cut-off values. CI = confidence interval, NPV = negative predictive value, PPV = positive predictive value.

Cut-off value	Sensitivity	95% CI	Specificity	95% CI
<-28.88	0.00	0.0 – 11.6	100.00	88.4 – 100.0
≤-26.36	6.67	0.8 – 22.1	96.67	82.8 – 99.9
≤-25.55	6.67	0.8 – 22.1	93.33	77.9 – 99.2
≤-22.30	13.33	3.8 – 30.7	93.33	77.9 – 99.2
≤-17.62	13.33	3.8 – 30.7	86.67	69.3 – 96.2
≤-16.42	20.00	7.7 – 38.6	86.67	69.3 – 96.2
≤-16.42	20.00	7.7 – 38.6	83.33	65.3 – 94.4
≤-15.22	26.67	12.,3 - 4.,9	83.33	65.3 – 94.4
≤-15.10	26.67	12.3 – 45.9	80.00	61.4 – 92.3
≤-15.09	30.00	14.7 – 49.4	80.00	61.4 – 92.3
≤-14.48	30.00	14.7 – 49.4	73.33	54.1 – 87.7
≤-12.80	36.67	19.9 – 56.1	73.33	54.1 – 87.7
≤-12.19	36.67	19.9 – 56.1	70.00	50.6 – 85.3
≤-8.41	63.33	43.9 – 80.1	70.00	50.6 – 85.3
≤-7.96	63.33	43.9 – 80.1	66.67	47.2 – 82.7
≤-7.19	73.33	54.1 – 87.7	66.67	47.2 – 82.7
≤-5.77	73.33	54.1 – 87.7	63.33	43.9 – 80.1
≤-4.26	80.00	61.4 – 92.3	63.33	43.9 – 80.1
≤-3.31	80.00	61.4 – 92.3	60.00	40.6 – 77.3
≤-3.23	83.33	65.3 – 94.4	60.00	40.6 – 77.3
≤-2.25	83.33	65.3 – 94.4	53.33	34.3 – 71.7
≤-1.97	86.67	69.3 – 96.2	53.33	34.3 – 71.7
≤-1.41	86.67	69.3 – 96.2	43.33	25.5 – 62.6
≤-0.23	93.33	77.9 – 99.2	43.33	25.5 – 62.6
≤0.74	93.33	77.9 – 99.2	40.00	22.7 – 59.4
≤1.28	96.67	82.8 – 99.9	40.00	22.7 – 59.4
≤8.41	96.67	82.8 – 99.9	20.00	7.7 – 38.6
≤8.86	100.00	88.4 – 100.0	20.00	7.7 – 38.6
≤86.90	100.00	88.4 – 100.0	0.00	0.0 – 11.6

Supplementary Table 6. Summary performance characteristics of small-volume ADC cut-off values. CI = confidence interval.

Absolute follow-up T2WI-derived maximum tumour diameter increase cut-off, mm	Number of progressors (%)	Number of non-progressors (%)
**Patients with an absolute follow-up increase in T2WI-derived maximum tumour diameter ≥ 1 mm**		
1	24 (61%)	15 (39%)
2	20 (65%)	11 (35%)
3	18 (72%)	7 (28%)
4	16 (80%)	4 (20%)
5	9 (82%)	2 (18%)
**Patients with a relative follow-up increase in T2WI-derived maximum tumour diameter ≥ 20%**		
1	17 (68%)	8 (32%)
2	16 (67%)	8 (33%)
3	16 (73%)	6 (27%)
4	15 (79%)	4 (21%)
5	9 (82%)	2 (18%)
**Patients with a relative follow-up decrease in small-volume ADC values ≤ -10%**		
1	12 (75%)	4 (25%)
2	10 (83%)	2 (17%)
3	9 (90%)	1 (10%)
4	9 (90%)	1 (10%)
5	4 (100%)	0 (0%)

Supplementary Table 7. The number of progressors and non-progressors among patients with different follow-up changes in T2WI-derived absolute and relative maximum tumour diameter and small-volume ADC values.
